# Microtensile bond strength of resin composite to dentin using different adhesive systems and directions of electric current

**DOI:** 10.1590/0103-6440202204870

**Published:** 2022-12-05

**Authors:** Maurício Bottene Guarda, Rafael Rocha Pacheco, Isaias Donizeti Silva, William Cunha Brandt, Mario Alexandre Coelho Sinhoreti, Rafael Pino Vitti

**Affiliations:** 1 State University of Campinas, Piracicaba Dental School, Piracicaba, SP, Brazil; 2 University of Detroit Mercy, School of Dentistry, Detroit, MI, USA; 3 Santo Amaro University, School of Dentistry, São Paulo, SP, Brazil; 4 Herminio Ometto University Center, School of Dentistry, Araras, SP, Brazil

**Keywords:** adhesion, bond strength, dentin, electrical current, resin composite

## Abstract

Thisstudy aimed to evaluate the effect of the electric current direction application on the resin composite-dentin bond strength using three adhesive systems. Human molar teeth were distributed according to the adhesive system (two-step self-etch - Clearfil SE Bond, Kuraray [CSE]; one-step self-etch - Single Bond Universal, 3M ESPE [SBU]; and two-step etch-and-rinse - Adper Single Bond 2, 3M ESPE [SB2]), electric current direction (without electric current - control, direct and reverse electric currents - 35µA), and storage time (24h - immediate and 6 months). Resin composite blocks (Filtek Z350XT, 3M ESPE) were bonded to dentin. The teeth/resin composites specimens were stored in distilled water at 37ºC for 24 hours and 6 months for the microtensile bond strength (µTBS) test (n = 10; ~12 sticks for each tooth). Failure patterns were analyzed on a stereomicroscope and classified as cohesive-dentin, cohesive-resin, adhesive or mixed. Adhesive penetration into dentin and hybrid layer formation were evaluated in a scanning electron microscope (n = 6). Data were submitted to a three-way ANOVA followed by Tukey’s *post hoc* test (α = 0.05). There are no differences in µTBS when the adhesive systems were applied under direct and reverse electric currents, but both electric currents increased the µTBS for all adhesive systems. SBU showed the lowest µTBS values for control groups in both storage times and direct electric current in 6 months of storage. The adhesive failure pattern was more frequently observed in all groups. The electric current formed long resin tags for all adhesive systems. Storage for 6 months did not significantly decrease µTBS values. Both directions of electric current (positive and negative charges) at 35µA can increase the µTBS of the adhesive systems tested to dentin.

## Resumo

O objetivo neste estudo foi avaliar o efeito da direção da corrente elétrica na resistência da união resina composta-dentina usando três sistemas adesivos. Dentes molares humanos foram distribuídos de acordo com o sistema adesivo (dois passos autocondicionante - Clearfil SE Bond, Kuraray [CSE]; e um passo autocondicionante - Single Bond Universal, 3M ESPE [SBU]; e dois passos convencional - Adper Single Bond 2, 3M ESPE [SB2]), a direção da corrente elétrica (sem corrente elétrica - controle, correntes elétricas direta e reversa - 35µA) e tempo de armazenamento (24h - imediato e 6 meses). Blocos de resina composta (Filtek Z350XT, 3M ESPE) foram aderidos à dentina. Amostras de dentina-resina foram produzidas e armazenadas em água destilada a 37ºC por 24 horas e 6 meses para o teste de resistência da união à microtração (µTBS) (n = 10; ~12 palitos por dente). Os padrões de fratura foram analisados ​​em estereomicroscópio e classificados em falhas coesiva na dentina, coesiva na resina, adesiva ou mista. A penetração do adesivo na dentina e a formação da camada híbrida foram avaliadas em microscópio eletrônico de varredura (MEV). Os dados foram submetidos à ANOVA três fatores seguidos pelo teste *post hoc* de Tukey (α = 0,05). Não houve diferenças na µTBS quando os sistemas adesivos foram aplicados sob as correntes elétricas direta e reversa, mas ambas as correntes elétricas aumentaram a µTBS para todos os sistemas adesivos. SBU apresentou os menores valores de µTBS para o grupo controle em ambos os tempos de armazenamento e para a corrente elétrica direta em 6 meses de armazenamento. Falhas adesivas foram mais frequente em todos os grupos. A corrente elétrica formou longos *tags* resinosos para todos os sistemas adesivos. O armazenamento por 6 meses não diminuiu significativamente os valores de µTBS. Ambos os sentidos da corrente elétrica (cargas positivas e negativas) a 35µA podem aumentar a µTBS dos sistemas adesivos testados à dentina.

## Introduction

Modern adhesive dentistry allows sound dental tissue preservation while still obtaining adequate retention of restorations (1). The quality of the adhesive layer determines the longevity and bond strength between the restorative material and dental substrates (2). Dentinal sealing ability (and consequent clinical success) is related to the hybrid layer extension (structure formed between adhesive system and dentin) and not specifically its thickness (2-5). Different adhesive techniques lead to different adhesive interfaces (3-5). Self-etch adhesive systems present reduced technique sensitivity since there is no need for etching dentin with phosphoric acid when compared to total-etch techniques and, consequently, no need for dentin moisture control (5).

Due to increased permeability, self-etch adhesive systems can lead to high nano-infiltration which can compromise the hybrid layer (6). On the other hand, total-etch adhesive systems promote an incomplete infiltration of monomers through collagen fibers in demineralized dentin. The exposed collagen network, non-permeated by the adhesive system, can lead to increased degradation of the hybrid layer (2-5). High content of hydrophilic monomers can lead to increased water sorption and degradation for simplified adhesive systems, where primer and bond are in one component. Also, high concentrations of solvent needed for the primer can reduce the degree of conversion and, consequently, the mechanical properties of the adhesive layer (7,8). The amount of monomer penetration into the collagen network is directly related to the adhesive layer quality (2-5).

Adhesive systems are physically applied to the tooth surface by using small disposable brushes (9,10). However, many technique modifications have been reported to increase dentinal sealing, including (but not limited to): the application of multiple coats of the adhesive system (11), application of one additional layer of a hydrophobic resin (12), increase of exposure time when light-curing the adhesive system (13), use of MMP inhibitors (such as chlorhexidine) (14), hot air blow and increased time of solvent evaporation (15), and active application (16). Some of these approaches lead to an increase in the number of steps for the dentin bonding procedure, which can increase technique sensitivity (5,17). The use of electric current can improve dentin wettability as well as dentin hybridization and bond strength, without increasing the number of clinical steps (18-21). The only difference for adhesive system application using electric current is the type of applicator that is used, with no influence on a number of clinical steps.

The electric current (from 5 to 50µA) is a safe adhesive system application mode to dentin, with better impregnation of these adhesive systems (18-21). In general, a positive charge is used to apply the adhesive systems onto the dentin and a negative charge is kept in contact with the root dentin (direct electric current) (18-21). Adhesive systems may contain polar resin components (HEMA, PENTA, BPDM, among others) that can interact with the electric field, leading to higher monomer infiltration into the demineralized collagen network (21,22). Increased dentin wettability is observed due to bio-physical modifications in the collagen network caused by the electric current (i.e., alterations in the three-dimensional collagen network and the nature of physicochemical interactions/bonds) (22). However, there are no studies about the effects of electric current direction (polarity) on the bond strength between adhesive systems and dentin.

Thus, this study aimed to evaluate the influence of different directions of electric current on the microtensile bond strength (µTBS) to dentin using total-etch and self-etch adhesive systems stored in water for 24 hours and 6 months. The null hypotheses tested are that [i] different electric current directions, [ii] the adhesive systems, and [iii] the water storage (6 months) do not influence the µTBS.

## Materials and methods

### Specimen preparation

Sound human third molars extracted for therapeutic reasons with completed root formation and no presence of caries and/or restorations were stored in distilled water at 4°C for utilization within 6 months after approval from the Ethics Committee (CAAE 51055215.0.0000.5418). Teeth were sectioned 1.5mm above the cementoenamel junction to obtain a flat and deep dentin surface using a low-speed water-cooled diamond saw (Isomet 1000, Buehler, Lake Bluff, IL, USA). Teeth with exposed pulp chamber were discarded. The coronal flat dentin surface was ground with wet 600-grit sandpaper for 30s to create a standardized smear layer. This in vitro study involved a 3x3x2 factorial design. The factors were: the type of adhesive system, application mode, and storage time. The minimum sample size was calculated as n=8 per group, based on data from pilot study as well as previous studies (18-20); µTBS of 32.16 (6.10) MPa in the conventional application (control group) and 44.57 (6.82) MPa in the electric current application groups; using $\alpha$ of 0.05 and power of 90%.

Specimens were randomly divided into groups (n=10) according to the adhesive system, application method, and storage. Materials' specifications and application methods are described in Box 1. Three adhesive systems were evaluated: two-step self-etch (CSE; Clearfil SE Bond, Kuraray, Okayama, Japan), two-step total-etch (SB2; Adper Single Bond 2, 3M ESPE, St. Paul, MN, USA), and one-step self-etch (SBU; Single Bond Universal, 3M ESPE). Then, specimens were placed on moist sponges to simulate the moisture of periodontal conditions and to create an electric circuit (18-20). Sponge-dentin specimen set was connected to the experimental electric current device. A disposable applicator brush ("microbrush") was coupled to the tip of the electric current device, and changed for each specimen. For the conventional application (control group), the adhesive systems were applied with the experimental device turned off (0µA). For the experimental groups (direct and reverse electric currents), prior to the application of adhesive systems, the electric current was set at 35µA. For the direct electric current (DC), the negative charge (cathode) and positive charge (anode) were attached to the sponge and the specimen, respectively, creating an electric circuit. For the reverse electric current (RC), the positive charge was attached to the sponge while the negative charge was attached to the specimen to create the electric circuit (Figure 1). Before SB2 application only, the dentin surface was etched using phosphoric acid at 35% (Ultra-Etch, Ultradent, South Jordan, UT, USA) for 15s. The application of adhesive systems followed manufacturers' recommendations (Box 1). Adhesive systems were light-activated for 20s using a multiple-emission peak light-curing unit (1,200mW/cm$^2$; Bluephase G2, Ivoclar Vivadent, NY, USA) kept as close as possible to dentin surface. The light irradiance was measured using a radiometer (RD-7, ECEL, Ribeirão Preto, SP, Brazil). Specimens were restored with two-2.0mm increments of nanofilled resin composite (Filtek Z350 XT, 3M ESPE). Each increment of resin composite was lightcured using the same light-curing unit used for adhesive systems. Specimens were stored in distilled water at 37°C for 24h or 6 months.

**Box 1.** Materials evaluated in this study.

| Material | pH | Composition | Application methods |
|---|---|---|---|
| Adper Single Bond 2 (SB2) | 4.7 | HEMA, Bis-GMA, Glycerol 1,3-dimethacrylate, Diurethane dimethacrylate, water, ethanol, photoinitiators, silanized silica, polyacrylic and itaconic acid copolymer. | Etching with phosphoric acid at 35% (pH = 0.6) for 15s, water rinsing for 10s, removal of water excess (keeping dentin moist), application of adhesive system for 30s, gentle air blow e light-curing for 10s. |
| Clearfil SE Bond (CSE) | 2.1 | Primer: 10-MDP, HEMA, hydrophilic dimethacrylates, CQ, N,N-diethanol-p-toluidine, water.<br>Bond: 10-MDP, bis-GMA, HEMA, hydrophilic dimethacrylates, CQ, N,N-diethanol-p-toluidine, coloidal silanized silica and N,N-diethanol-p-toluidine. | Active application of primer for 20s, gentle air blow, application of bond, gentle air blow and light-curing for 10s. |
| Single Bond Universal (SBU) | 2.7 | HEMA, MDP, dimethacrilates, polyalkenoic acid, filler particles, ethanol, water, CQ, silane. | Active application of adhesive system for 20s, gentle air blow for 5s and light-curing for 10s. |
| Filtek Z350 XT | - | Bis-GMA, bis-EMA, UDMA, TEGDMA, silica/zirconia nanoparticles (20nm), nanoclusters of silica/zirconia nanoparticles (5-20nm) in a total of 78.5%wt. | Incremental technique, using increments of 2mm, and light-curing each increment for 10s |

Abbreviations: HEMA: (hydroxyethyl) methacrylate; Bis-GMA: Bisphenol A glycidylmethacrylate; MDP: methacryloyloxydecyl dihydrogen phosphate; CQ: camphorquinone; UDMA: urethane dimethacrylate; TEGDMA: triethylene glycol dimethacrylate.

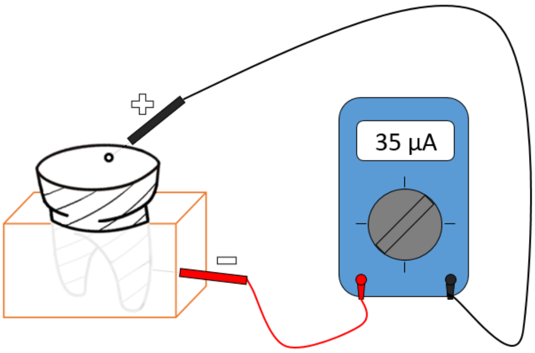

**Figure 1.** Schematic illustration of the adhesive systems applied on dentin using electric current.

### Microtensile bond strength (µTBS)

Each specimen was sectioned to obtain dentin-resin composite rectangular bar-shaped stick (~1mm$^2$) using a low-speed water-cooled diamond saw (Isomet 1000, Buehler). Adhesive area was measured using a digital caliper (Mitutoyo, Tokyo, Japan). Each stick was analyzed in a stereomicroscope (50x; MZ75, Leica Microsystems, Wetzlar, Germany) to verify any possible defects occurred during specimen cutting. Any defective stick was discarded. Sticks (~12 for each tooth) were stored in distilled water for 24h at 37°C.

For µTBS test, sticks were attached to jigs using cyanoacrylate glue (Loctite Super Bonder Power Flex Gel, Henkel, Rocky Hill, NY, USA). Sticks were tested until failure in a universal testing machine (Instron 1144, Canton, MA, USA) using a 500N load cell at 0.5mm/min crosshead speed until failure. The adhesive area was measured ($mm^2$) with a digital caliper (Mitutoyo). The µTBS was expressed in MPa following the equation: µTBS = F/A in which F is the force applied during the test (N) and A is the specimen bonded area ($mm^2$).

Failure patterns were determined by using an optical microscope (MZ75; Leica Microsystems, Heerbrugg, Switzerland) with a magnification of 50x, and classified as: cohesive in dentin, cohesive in resin, adhesive (cohesive in adhesive or at adhesive interface), or mixed.

The µTBS data were assessed for normality with the Shapiro-Wilk test and for homogeneity of variance with Levene's test. Results were submitted to three-way ANOVA (adhesive system, application mode, and storage) followed by Tukey's *post hoc* test with a pre-set alpha of 0.05.

**Scanning electron microscopy (SEM)**

Teeth (n=6) were sectioned to separate the root from the cusp, a flat dentin surface was obtained, and the adhesive systems were applied to dentin as previously described (Box 1). After restoration with two-2.0mm increments of nanofilled resin composite (Filtek Z350 XT, 3M ESPE), specimens were sectioned into 1.0mm-thick slices in the medium third. Slices were embedded in epoxy resin and wet-polished using 200-, 600-, 2000-, and 4000-grit sandpaper for 60s followed by diamond polishing pastes (1.0 and 0.5 µm; Ultradent Diamond Polish Mint, Ultradent Products Inc., South Jordan, UT, USA) with felt disks. After polishing, specimens were cleaned by sonication in distilled water for 5 min, demineralized in 6 mol/L HCl for 30s to remove minerals not protected by resin monomer, and deproteinized in 1% NaOCl for 10 min to dissolve exposed collagen beneath the hybrid layer. The specimens were dehydrated in ascending concentrations of ethanol (50%, 60%, 70%, 80%, 90%, and 100%; 10 min each). Thus, specimens were mounted in aluminum stubs, and sputter coated with gold using a Bal-Tec Model SCD-050, Liechtenstein Metallizer). Then, specimens were analyzed in scanning electron microscopy (SEM; JSM-5600LV, JEOL, Tokyo, Japan), operated under 15kV in different magnifications (x500, x1000, and x2000).

## Results

The 3-way ANOVA showed that there was no significant interaction between the three factors evaluated (adhesive × electric current × time; ρ=0.97826), between the adhesive × time factors (ρ=0.60386), electric current × time (ρ=0.57045) and neither for the time factor alone (ρ=0.78903) (Table 1.). The interaction adhesive × electric current (ρ=0.00281) and the isolated factors adhesive (ρ=0.00001) and electric current (ρ=0.00001) showed statistical significance.

**Table 1.** µTBS mean (±SD) values (MPa) for evaluated adhesive systems applied with different application mode for 24 hours and 6 months of water storage.

| Groups | 24 hours | | | 6 months | | |
|---|---|---|---|---|---|---|
| | CSE | SBU | SB2 | CSE | SBU | SB2 |
| Control | 43.7 (4.3) | 28.2 (6.6) | 39.4 (7.4) | 42.8 (7.6) | 29.0 (8.3) | 37.5 (5.9) |
| DC | 52.3 (7.2) | 48.1 (6.5) | 50.2 (7.1) | 51.0 (6.3) | 43.7 (5.1) | 49.7 (6.3) |
| RC | 53.1 (6.6) | 47.5 (5.4) | 50.7 (6.4) | 50.5 (5.4) | 45.8 (6.0) | 48.2 (5.7) |

For adhesive systems, the lowest µTBS values were observed for SBU at 24h in conventional (control) application, and at 6 months for control and DC groups. For electric current, DC and RC groups showed the highest µTBS values for all adhesive systems tested at 24h and 6 months. No differences were observed for µTBS between storage times (ρ=0.78903).

For all adhesive systems tested, the most common failure pattern observed was adhesive, followed by mixed failures (CSE and SB2). For SBU, the second most common failure observed was the cohesive in dentin (Figure 2).

It is possible to observe an improvement of dentin sealing, with larger resin tags as well as thicker and more homogeneous hybrid layer obtained by electric current application (Figure 3). However, this result was adhesive system-dependent. For CSE/control (Figure 3 - 1A) it is possible to observe shorter resin tags compared to CSE/DC (Figure 3 - 1B) and CSE/RC (Figure 3 - 1C). For SBU/control (Figure 3 -

2A) it is possible to observe small resin tag formation when compared to SBU/DC and SBU/RC that showed resin tags more closely adapted to dentin (Figure 3 - 2B and 2C). For SB2/control (Figure 3 - 3A) it is possible to observe a reduced number and size of resin tags when compared to SB2/DC and SB2/RC (Figure 3 - 3B and 3C).

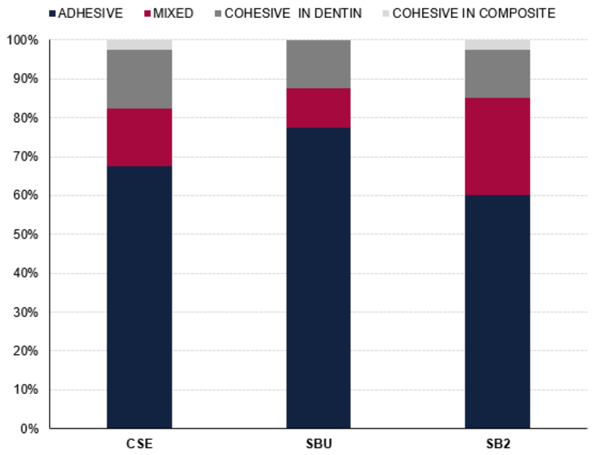

**Figure 2**. Failure patterns observed at 50x magnification for evaluated adhesive systems.

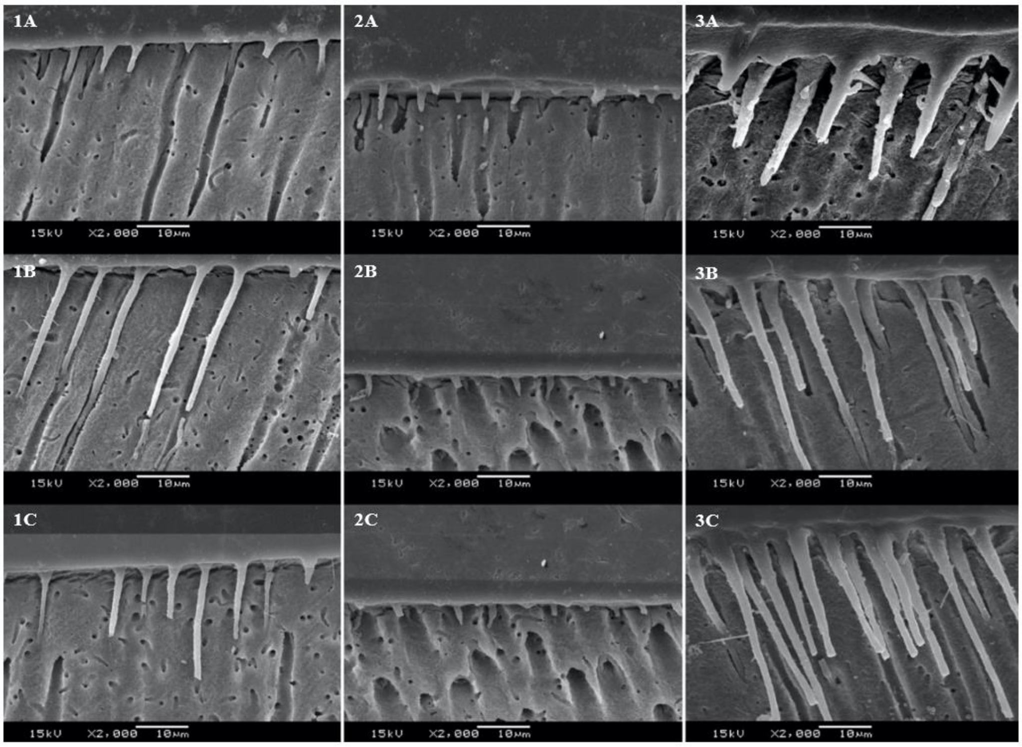

**Figure 3.** SEM images. 1A: CSE/control; 1B: CSE/DC; 1C: CSE/RC; 2A: SBU/control; 2B: SBU/DC; 2C: SBU/RC; 3A: SB2/control; 3B: SB2/DC; 3C: SB2/RC.

## Discussion

It is known that DC application of adhesive systems can increase its impregnation to dentin (18-21). Studies have shown that electric current direction (DC or RC) can influence the results in some situations (23-26). Thus, it is important to evaluate if the direction of electric current (polarity) influences the adhesion of resin composites to dentin. In this study, the direction of the electric current did not influence adhesion. Thus, the first hypothesis was accepted since different direction of the electric currents did not influence the µTBS values (Table 2).

Resin monomers can be attracted by an electric current, increasing its flow into demineralized dentin (18-20). Electric current causes dielectric dispersion in tooth, improving ion diffusion and interfacial polarization (22). Furthermore, the electric current breaks the surface tension of resin monomers with high molecular weight and changes the molecular arrangement of resin monomers, increasing its viscosity and flexibility (27). The application of adhesive systems using electric current (DC and RC groups) was able to improve the formation of resin tags, resulting in a more homogeneous hybrid layer, with efficient infiltration of resin monomers and, consequently, a longer and higher number of resin tags (Figure 2). The low superficial tension of the adhesives associated with an increase on dentinal surface energy after electric current application improved the adhesive system penetration into dentin. Furthermore, the removal of excess water and solvent is not ideal during the conventional "solvent evaporation" step (gentle air blow). DC and RC electric current currents could have improved the substitution rate of water by resin monomers, as well as it might favor solvent evaporation and improve the hybrid layer quality (20). These parameters can explain the highest µTBS values when adhesive systems were applied under DC and RC. In addition, the current direction did not affect these parameters.

**Table 2.** µTBS mean (±SD) values (MPa) for evaluated adhesive systems applied with different application mode.

| Groups | CSE | SBU | SB2 |
|---|---|---|---|
| Control | 43.3 (6.0) Ab | 28.6 (7.5) Bb | 38.5 (6.7) Ab |
| DC | 51.7 (6.8) Aa | 45.9 (5.8) Ba | 50.0 (6.7) ABa |
| RC | 51.8 (6.0) Aa | 46.7 (5.7) Aa | 49.5 (6.1) Aa |

Different letters indicate statistically significant difference: capital letters for comparison between adhesive system (rows) and lowercase letters for comparison between application mode (columns) ($p<0.05$).

The second hypothesis was rejected since statistical differences in µTBS values were observed among adhesive systems (Table 2). Differences in the composition of these materials (Box 1) could explain this result. CSE contains a high concentration of hydrophilic polar monomers (27), and these monomers are more influenced by the electric current (18,19). Also, CSE possesses an acidic monomer with a slightly lower pH when compared to SBU. An increased pH in this case could lead to reduced dentin demineralization and consequently reduced penetration (24). The hydrophilic nature of some of these monomers can lead to a "permeable-membrane behavior" of the adhesive layer, which can lead to water absorption even when polymerized (25). This effect can lead to plasticization of the resin matrix decreasing the µTBS (28). Electric current may facilitate the diffusion of the adhesive with polar monomers into dentin (20). Adhesive systems with high amount of polar monomers are more influenced by the electric current.

The resin composite/dentin adhesive interface is susceptible to hydrolytic degradation, which can affect the µTBS (2,29). Slow water absorption by chemical reagents of the adhesive systems can contribute to hydrolytic degradation of the adhesive interface by water molecule diffusion into this zone (4). However, the third tested hypothesis was accepted since no differences were observed for µTBS between different storage times. Increased monomer infiltration into dentin should not be related exclusively to an increase in dentin permeability, but also an increased monomeric mobility (6). Electric currents can also influence wetting of dental substrates, rate of water displacement by adhesive systems (22), and facilitate the infiltration of adhesive into demineralized dentin by iontophoresis force onto the polar monomers, resulting in a more complete infiltration of dentin (29) (Figure 2). Polar monomers are oriented through the exposed collagen fibrils, creating a more compact hybrid layer (23). Thus, DC and RC groups may support dentin demineralization and resin monomer infiltration, creating a stronger and more stable hybrid layer (3,19). According to previous studies (19,21), it is hypothesized that the application of adhesive systems using DC and RC electric currents is important to form a stronger, more compact, and stable hybrid layer, creating a resin composite/dentin adhesive interface less prone to hydrolytic degradation. Despite the results of the present study showing stable µTBS for DC and RC groups, similar results were found for control groups. These results must be carefully analyzed, since 6 months of storage is a short time to observe a decrease in µTBS (19).

The present study obtained positive results in the infiltration of the resin monomers applied with direct and reverse electric currents on dentin, increasing and stabilizing the µTBS (clinically relevant). This procedure could be used clinically, since DC and RC electric current can be safely applied without adverse effects (18-21) with a similar operation/device to a pulp tester/apex locator (21).

Although the electric current provides an increase in bonding performance for the adhesive systems tested, it is noteworthy that such results were obtained from DC and RC electric currents set at 35μA. Different intensities of electric current could show different bonding performances because electric currents modify the dentin biophysical and biochemical properties (21). Therefore, further studies evaluating the effects of different electric currents and other factors (different adhesive systems, longer storage times, and other intensities of electric current, among others) are important to corroborate with the results of the present study.

## Resumo

O objetivo neste estudo foi avaliar o efeito da direção da corrente elétrica na resistência da união resina composta-dentina usando três sistemas adesivos. Dentes molares humanos foram distribuídos de acordo com o sistema adesivo (dois passos autocondicionante - Clearfil SE Bond, Kuraray [CSE]; e um passo autocondicionante - Single Bond Universal, 3M ESPE [SBU]; e dois passos convencional - Adper Single Bond 2, 3M ESPE [SB2]), a direção da corrente elétrica (sem corrente elétrica - controle, correntes elétricas direta e reversa – 35μA) e tempo de armazenamento (24h - imediato e 6 meses). Blocos de resina composta (Filtek Z350XT, 3M ESPE) foram aderidos à dentina. Amostras de dentina-resina foram produzidas e armazenadas em água destilada a 37ºC por 24 horas e 6 meses para o teste de resistência da união à microtração (μTBS) (n = 10; ~12 palitos por dente). Os padrões de fratura foram analisados em estereomicroscópio e classificados em falhas coesiva na dentina, coesiva na resina, adesiva ou mista. A penetração do adesivo na dentina e a formação da camada híbrida foram avaliadas em microscópio eletrônico de varredura (MEV). Os dados foram submetidos à ANOVA três fatores seguidos pelo teste *post hoc* de Tukey ($\alpha$ = 0,05). Não houve diferenças na μTBS quando os sistemas adesivos foram aplicados sob as correntes elétricas direta e reversa, mas ambas as correntes elétricas aumentaram a μTBS para todos os sistemas adesivos. SBU apresentou os menores valores de μTBS para o grupo controle em ambos os tempos de armazenamento e para a corrente elétrica direta em 6 meses de armazenamento. Falhas adesivas foram mais frequente em todos os grupos. A corrente elétrica formou longos *tags* resinosos para todos os sistemas adesivos. O armazenamento por 6 meses não diminuiu significativamente os valores de μTBS. Ambos os sentidos da corrente elétrica (cargas positivas e negativas) a 35μA podem aumentar a μTBS dos sistemas adesivos testados à dentina.
